# Cross Entropy in Deep Learning of Classifiers Is Unnecessary—ISBE Error Is All You Need

**DOI:** 10.3390/e26010065

**Published:** 2024-01-12

**Authors:** Władysław Skarbek

**Affiliations:** Faculty of Electronics and Information Technology, Warsaw University of Technology, 00-661 Warszawa, Poland; wladyslaw.skarbek@pw.edu.pl

**Keywords:** deep learning, cross entropy, normalization function, neural network, model inference, gradient backpropagation

## Abstract

In deep learning of classifiers, the cost function usually takes the form of a combination of SoftMax and CrossEntropy functions. The SoftMax unit transforms the scores predicted by the model network into assessments of the degree (probabilities) of an object’s membership to a given class. On the other hand, CrossEntropy measures the divergence of this prediction from the distribution of target scores. This work introduces the ISBE functionality, justifying the thesis about the redundancy of cross-entropy computation in deep learning of classifiers. Not only can we omit the calculation of entropy, but also, during back-propagation, there is no need to direct the error to the normalization unit for its backward transformation. Instead, the error is sent directly to the model’s network. Using examples of perceptron and convolutional networks as classifiers of images from the MNIST collection, it is observed for ISBE that results are not degraded with SoftMax only but also with other activation functions such as Sigmoid, Tanh, or their hard variants HardSigmoid and HardTanh. Moreover, savings in the total number of operations were observed within the forward and backward stages. The article is addressed to all deep learning enthusiasts but primarily to programmers and students interested in the design of deep models. For example, it illustrates in code snippets possible ways to implement ISBE functionality but also formally proves that the SoftMax trick only applies to the class of dilated SoftMax functions with relocations.

## 1. Introduction

A deep model is a kind of mental shortcut [1], broadly understood as a model created in deep learning of a certain artificial neural network N , designed for a given application. What, then, is an artificial neural network [2], its deep learning [3,4], and what applications [5] are we interested in?

From a programmer’s perspective, an artificial neural network is a type of data processing algorithm [6], in which subsequent steps are carried out by configurable computational units, and the order of processing steps is determined by (dynamically created) computing graph. The computing graph is always directed and acyclic (DAG). Interestingly, even recurrent neural networks, such as the LSTM (Long Short-Term Memory) nets, which are trained using gradient methods, have DAG-type computational graphs defined.

At the training stage, each group of input data *X*, i.e., each group of training examples, technically each batch of training examples, first undergoes the *inference (forward)* phase on the current model, i.e., processing through the network N at its current parameters *W*. As a result, network outputs $Y\leftarrow F_{N}\left(X;W\right)$ are produced [7]. The result of the entire network N with the functionality $F_{N}$ and joined set of parameters *W* is the result of combining the results of the activities for individual units U with individual functionalities $F_{U}$ and with possible individual parameters $W_{U}$ , as well.

$$\begin{array}{c} \overset{\mathrm{Inference}}{\overset{︷}{\overset{X;\;W}{\to }\begin{array}{c} F_{N} \end{array}\overset{Y\leftarrow F_{N}\left(X;W\right)}{\to }}}\;\equiv \;\overset{\mathrm{Inference}}{\overset{︷}{\begin{array}{c} \;\cdots \;\overset{X_{u};\;W_{u}}{\to }\begin{array}{c} F_{U} \end{array}\overset{Y_{u}\leftarrow F_{U}\left(X_{u};W_{u}\right)}{\to }\;\cdots \; \end{array}}} \end{array}$$

After the inference phase comes the model update phase, where the current model is modified (improved) according to the selected optimization procedure [8]. *The model update phase* begins with calculating the loss (cost) value $Z\leftarrow L(Y,Y^{\circ })$ defined by the chosen loss function L as well as the inference outcome *Y* and the target result $Y^{\circ }$ .

$$\begin{array}{c} \overset{\mathrm{Inference}}{\overset{︷}{\overset{X;\;W}{\to }\begin{array}{c} F_{N} \end{array}\overset{Y\leftarrow F_{N}\left(X;W\right)}{\to }}}\;\cdots \;\overset{\mathrm{Model}\;\mathrm{update}\text{-}\mathrm{start}}{\overset{︷}{\overset{Y,Y^{\circ }}{\to }\begin{array}{c} L \end{array}\overset{Z\leftarrow L(Y,Y^{\circ })}{\to }}} \end{array}$$

The loss *Z* depends (indirectly through *Y*) on all parameters *W* and what conditions the next step of the update phase is the determination of sensitivity $\bar{W}$ of the loss function L to their changes. The mathematical model of sensitivity is the gradient $\bar{W}≐\frac{\partial L}{\partial W}$ . Knowing this gradient, the optimizer will make the actual modification of *W* in a direction that also takes into account the values of gradients obtained for previous training batches.

Calculating the gradient with respect to parameters actually assigned to different computational units required the development of an efficient algorithm for its propagation in the opposite direction to inference [9,10].

Just as in the inference phase, each unit U has its formula $Y_{u}\leftarrow F_{U}\left(X_{u},W_{u}\right)$ for processing data from input $X_{u}$ to output $Y_{u}$ with parameters $W_{u}$ , so in the *backward gradient propagation phase*, it must have a formula ${\bar{X}}_{u},{\bar{W}}_{u}\leftarrow {\bar{F}}_{U}\left({\bar{Y}}_{u}\right)$ for transforming the gradients assigned to its outputs ${\bar{Y}}_{u}$ into gradients assigned to its inputs ${\bar{X}}_{u}$ and its parameters ${\bar{W}}_{u}$ .

$$\begin{array}{c} \overset{\mathrm{BackPropagation}}{\overset{︷}{\overset{\bar{X};\;\bar{W}\leftarrow {\bar{F}}_{N}\left(\bar{Y};X,Y,W\right)}{\leftarrow }\begin{array}{c} {\bar{F}}_{N} \end{array}\overset{\bar{Y}}{\leftarrow }}}\;\cdots \;\overset{\mathrm{Loss}\;\mathrm{function}\;\mathrm{gradient}\;\frac{\partial L}{\partial Y}}{\overset{︷}{\overset{\bar{Y}\leftarrow \bar{L}\left(\bar{Z};Y,Z\right)}{\leftarrow }\begin{array}{c} \bar{L} \end{array}\overset{1=\frac{\partial Z}{\partial Z}}{\leftarrow }}}\\ \overset{\mathrm{Gradient}\;\mathrm{BackPropagation}}{\overset{︷}{\begin{array}{c} \;\cdots \;\overset{{\bar{X}}_{u};\;{\bar{W}}_{u}\leftarrow {\bar{F}}_{U}\left({\bar{Y}}_{u};X_{u},Y_{u},W_{u}\right)}{\leftarrow }\begin{array}{c} {\bar{F}}_{U} \end{array}\overset{{\bar{Y}}_{u}}{\leftarrow }\;\cdots \; \end{array}}} \end{array}$$

Based on such local rules of gradient backpropagation and the created computation graph, the *backpropagation* algorithm can determine the gradients of the cost function with respect to each parameter in the network. The computation graph is created during the inference phase and is essentially a stack of links between the arguments and results of calculations performed in successive units [10,11].

Deep learning is precisely a concert of these inference and update phases in the form of gradient propagation, calculated for randomly created groups of training examples. These phases, intertwined, operate on multidimensional, deep tensors (arrays) of data, processed with respect to network inputs, and on deep tensors of gradient data, processed with respect to losses, determined for the output data of the trained network.

Here, by a deep tensor, we mean a multidimensional data array that has many feature maps, i.e., its size along the feature axis is relatively large, e.g., 500, which means 500 scalar feature maps. We then say that at this point in the network, our data has a *deep representation* in a 500-dimensional space.

As for the applications we are interested in this work, the answer is those that have at least one requirement for classification [12]. An example could be crop detection from satellite images [13], building segmentation in aerial photos [14], but also text translation [15]. Classification is also related to voice command recognition [16], speaker recognition [17], segmentation of the audio track according to speakers [18], recognition of speaker emotions with visual support [19], but also classification of objects of interest along with their localization in the image [20].

It may be risky to say that after 2015, in all the aforementioned deep learning classifiers, the cost function takes the form of a composition of the SoftMax function [21] and the CrossEntropy function, i.e., cross-entropy [22]. The SoftMax unit normalizes the scores predicted by the classifier model for the input object into *SoftMax* scores that sum up to one, which can be treated as an estimation of the conditional probability distribution of classes. Meanwhile, cross-entropy measures the divergence of this estimation from the target probability distribution (class scores). In practice, the target score may be taken from a training set prepared manually by a so-called teacher [23] or may be calculated automatically by another model component, e.g., in the knowledge distillation technique [24].

For *K* classes and $n_{b}$ training examples, the SoftMax function is defined for the raw score matrix $X\in R^{n_{b}\times K}$ as:

$$\left[Y\leftarrow SoftMax(X)\right]\;⟶\;\left[Y_{bi}\leftarrow \frac{e^{X_{bi}}}{\sum_{j\in [K]}e^{X_{bj}}},\;b\in \left[n_{b}\right],\;i\in \left[K\right]\right]\;,$$

where the notation [K] denotes any *K*-element set of indices—in this case, they are class labels.

The CrossEntropy function on the matrix $Y,Y^{\circ }\in R^{n_{b}\times K}$ is defined by the formula:

$$\left[Z\leftarrow CrossEntropy(Y,Y^{\circ })\right]⟶\left[Z_{b}\leftarrow -\sum_{j\in [K]}Y_{bj}^{\circ }\log_{e}Y_{bj},\;b\in \left[n_{b}\right],\;z\in R^{n_{b}}\right]$$

(1) $$\overset{\mathrm{Classifier}\;\mathrm{loss}\;\text{function}:\;\mathrm{Separated}\;\mathrm{Implementation}}{\overset{︷}{\begin{array}{c} \overset{\mathrm{Scores}\;\mathrm{Inference}}{\overset{︷}{\overset{\mathrm{classified}\;\mathrm{object}}{\to }\begin{array}{c} F_{N} \end{array}\overset{\mathrm{raw}\;\mathrm{scores}\;X}{\to }}}\\ \overset{\mathrm{Loss}\;\mathrm{Estimation}\;L}{\overset{︷}{\overset{\mathrm{raw}\;\mathrm{scores}\;X}{\to }\begin{array}{c} \mathrm{SoftMax} \end{array}\overset{\mathrm{soft}\;\mathrm{scores}\;Y,\;Y^{\circ }}{\to }\begin{array}{c} \mathrm{CrossEntropy} \end{array}\overset{\mathrm{losses}\;Z}{\to }}} \end{array}}}$$

When classifiers began using a separated implementation of the combination of the SoftMax normalization and the CrossEntropy loss, it quickly became evident in practice that its implementation had problems with scores close to zero, both in the inference phase and in the backward propagation of its gradient. In formulas of properties 1–3 in Theorem 1 of Section 2, we see from where the problem comes. Only the integration of CrossEntropy with normalization SoftMax eliminated these inconveniences. The integrated approach has the following form:

$$\overset{\mathrm{Classifier}\;\mathrm{loss}\;\mathrm{function}\text{-}\mathrm{Integrated}\;\mathrm{Implementation}}{\overset{︷}{\begin{array}{c} \overset{\mathrm{Inference}}{\overset{︷}{\overset{\mathrm{classified}\;\mathrm{object}}{\to }\begin{array}{c} F_{N} \end{array}\overset{\mathrm{raw}\;\mathrm{scores}\;X}{\to }}}\\ \overset{\mathrm{Loss}\;\mathrm{Estimation}\;L}{\overset{︷}{\overset{\mathrm{raw}\;\mathrm{scores}\;X,\mathrm{soft}\;\mathrm{scores}\;,Y^{\circ }}{\to }\begin{array}{c} \mathrm{CrossEntropy}\;\circ \;\mathrm{SoftMax} \end{array}\overset{\mathrm{losses}\;Z}{\to }}} \end{array}}}$$

The integrated functionality of these two features has the following redundant mathematical notation:

$$\begin{array}{c} Z\leftarrow \left[CrossEntropy\;\circ \;SoftMax\right]\left(X,Y^{\circ }\right)⟶\\ Z_{b}\leftarrow -\sum_{j\in [K]}Y_{bj}^{\circ }\log_{e}\frac{e^{X_{bj}}}{\sum_{i\in [K]}e^{X_{bi}}},\;b\in \left[n_{b}\right] \end{array}$$

This redundancy in notation was helpful in deriving the equation for the gradient backpropagation for the integrated loss function CrossEntropy∘SoftMax . Later, we will also use for such composition the name SoftCE .

The structure of this paper is as follows:

1. The second section is devoted to mathematics of the *SoftMax trick*. Its validity is proved in two ways:
  (a) Using gradient formulas for the composition of differentiable functions;
  (b) Using the concept of the Jacobian matrix and linear algebra calculus.
2. In the third section titled *ISBE Functionality*, the conditions that a normalization unit must meet for its combination with a cross-entropy unit to have a gradient at the input equal to the difference in soft scores: $\bar{X}=Y-Y^{\circ }$ are analyzed. Then the definition of ISBE functionality is introduced, which in the inference phase (I) normalizes the raw score to a soft score (S), and in the backward propagation phase (B) returns an error (E), equal to the difference in soft scores. It is also justified why, in the case of the SoftMax normalization function, the ISBE functionality has, from the perspective of the learning process, the functionality of the integrated element CrossEntropy ∘ SoftMax.
3. In the first subsection of the fourth section, using the example of the problem of recognizing handwritten digits and the standard MNIST(60K) image collection [25], numerous experiments show that in addition to the obvious savings in computational resources, in the case of five activations serving as normalization functions, the classifier’s effectiveness is not lower than that of the combination of the normalization SoftMax and Cross Entropy. This ISBE property was verified for the activation units SoftMax, Sigmoid, Hardsigmoid, and Tanh and Hardtanh. The second subsection of the fourth section reports on how ISBE behaves for a more demanding dataset CIFAR-10 and a more complex architecture VGG-16.
4. The final fifth section contains conclusions.
5. In Appendix A, the class of functions leading to the dilated *SoftMax trick* is fully characterized using concepts of dilation and relocation of function domain.
6. In Appendix B the ISBE functionality is integrated with PyTorch class torch.autograd. Function.

The main contributions of this research are:

1. Introducing ISBE functionality as simplification and, at the same time, extension of the functional combination of SoftMax with CrossEntropy.
2. Verification of ISBE feasibility and efficiency on two datasets and three CNN architectures.
3. Enhancement of theoretical background for the concept of *SoftMax trick* via its generalization and full characterization of normalization functions which exhibit this property.

Concluding this introduction, I would like to emphasize that this work is not intended to depreciate the concept of entropy in the context of machine learning. It has played and continues to play a key role as a loss function. Its form appears naturally in many data modeling tasks. For example, in the case of multi-class logistic regression, when computing optimal weights, maximizing the negative logarithm of the likelihood function directly leads to the cross-entropy function. In the context of modeling, cross-entropy will remain an important research tool. The context of the ISBE functionality concerns only the specific method of calculating the gradient of network parameters for the needs of SGD (Stochastic Gradient Descent) optimizers. Only this, nothing more.

## 2. Discrete Cross-Entropy and the SoftMax Trick Property

This section is devoted to the mathematics of the *SoftMax trick*. Its validity will be proved in two ways:

1. Using gradient formulas for the composition of differentiable functions,
2. Using the concept of Jacobian matrix and linear algebra calculus.

The discrete cross-entropy function CE of a target discrete probability distribution $y^{\circ }\in {\left[0,1\right]}^{K},\;\sum_{i}y_{i}^{\circ }=1$ , relative to the calculated by the classifier the probability distribution $y\in {\left(0,1\right)}^{K},$ $\sum_{i}y_{i}=1$ , is defined by the formula:

(2) $$CE\left(y^{\circ },y\right)≐-\sum_{i\in [K]}y_{i}^{\circ }\log_{e}y_{i}$$

The notation [K] refers to a sequence of *K* indexes, such as (1,…,K) or (0,1,…,K−1) .

It appears that the gradient of the cross entropy $CE(y^{\circ },y)$ with respect to *y* is not defined at the zero components:

(3) $$\nabla_{y}CE\left(y^{\circ },y\right)=-y^{\circ }÷y\;,$$

where the operation ÷ for the expression w=u÷v , denotes the division of vector *u* components by the components of vector *v*, i.e., $w_{i}≐u_{i}/v_{i}$ , i∈[K] .

In the special case when the vector $y\in {\left(0,1\right)}^{K}$ is calculated based on the vector $x\in R^{K}$ according to the formula $y_{i}=SoftMax{\left(x\right)}_{i}=e^{x_{i}}/\sum_{k}e^{x_{k}}$ , the gradient of CE with respect to *x* has a particularly simple resultant formula. Its simplicity was the reason for the term *SoftMax trick*:

(4) $$\begin{array}{c} y\left(x\right)≐y≐SoftMax\left(x\right),\;SoftCE\left(y^{\circ },x\right)≐CE\left(y^{\circ },SoftMax\left(x\right)\right)\\ ⟶\nabla_{x}SoftCE\left(y^{\circ },x\right)\overset{SoftMax\;trick}{=}y-y^{\circ } \end{array}$$

Some authors [26] also use the term *SoftMax trick* for that part of the proof showing that the derivative of the natural logarithm of the sum of functions $e^{x_{i}}$ equals to the SoftMax function.

The *SoftMax trick* can be described as a theorem and proved in two ways: the elementary one and via the matrix calculus. The following theorem includes elementary properties of the cross-entropy function, optionally preceded by the SoftMax normalization.

**Theorem** **1.** *Let $y^{\circ }\in {\left[0,1\right]}^{K},\;\sum_{k\in [K]}y_{k}^{\circ }=1,$ be the target probability distribution and y∈(0,1), $\sum_{k\in [K]}y_{k}=1,$ be the predicted probability distribution. Then*

 *1.* *For any i∈[K], $\frac{\partial CE(y^{\circ },y)}{\partial y_{i}}=\frac{y_{i}^{\circ }}{y_{i}}$ .*
 *2.*
$\nabla_{y}CE\left(y^{\circ },y\right)=y^{\circ }÷y≐{\left[\frac{y_{1}^{\circ }}{y_{1}},\ldots ,\frac{y_{K}^{\circ }}{y_{K}}\right]}^{⊺}$
 *3.* *The range of CE covers the positive part of the real axis:*
$$\left\{CE\left(y^{\circ },y\right):y^{\circ }\in {\left[0,1\right]}^{K},\;y\in {\left(0,1\right)}^{K}\right\}=\left(0,\infty \right)$$
 *4.* *Let $x\in R^{K}$ be the vector of raw scores, and $y^{\circ }$ be the target probability distribution. Then SoftMax normalization followed by CE , is defined as follows*
$$SoftCE\left(y^{\circ },x\right)≐-\sum_{j\in [K]}y_{j}^{\circ }\log_{e}\frac{e^{x_{j}}}{\sum_{k\in [K]}e^{x_{k}}}\;.$$
  *Contrary to CE only, SoftCE exhibits the bounded gradient:*
   *(a)* *The Jacobian of SoftMax equals to:*
$$Jacobian\left(SoftMax\right)\left(x\right)=\frac{\partial SoftMax(x)}{\partial x}=\mathrm{diag}\left[y\right]-yy^{⊺}\;where\;\;y=SoftMax\left(x\right)\;.$$
   *(b)* *For any i∈[K], $\frac{\partial \left[SoftCE\left(y^{\circ },x\right)\right]}{\partial x_{i}}=y_{i}-y_{i}^{\circ }$ , where $y_{i}={\left(SoftMax\left(x\right)\right)}_{i}$ .*
   *(c)* *
$\nabla_{x}SoftCE\left(y^{\circ },x\right)=y-y^{\circ }$ , where y=SoftMax(x) .*
   *(d)* *The range of SoftCE covers the interval (−1,1):
*
$$\left\{SoftCE\left(y^{\circ },x\right):y^{\circ }\in {\left[0,1\right]}^{K},\;x\in R^{K}\right\}=\left(-1,+1\right)$$

**Elementary** **proof** **of** **SoftCE** **properties.**
$$\begin{array}{c} y_{i}≐\frac{e^{x_{i}}}{\sum_{k}e^{x_{k}}}⟶\frac{\partial \left[\log_{e}\left(\sum_{k}e^{x_{k}}\right)\right]}{\partial x_{i}}=y_{i}⟶\\ \begin{array}{ccc} \frac{\partial SoftCE(y^{\circ },x)}{\partial x_{i}} & = & \frac{\partial \left[\sum_{j}y_{j}^{\circ }\log_{e}\left(\sum_{k}e^{x_{k}}\right)-\sum_{j}y_{j}^{\circ }\overset{x_{j}}{\overset{︷}{\log_{e}e^{x_{j}}}}\right]}{\partial x_{i}}\\ & = & y_{i}\overset{=1}{\overset{︷}{\sum_{j}y_{j}^{\circ }}}-y_{i}^{\circ }=y_{i}-y_{i}^{\circ } \end{array} \end{array}$$

   □

**Proof** **of** ***SoftCE*** **property** **using** **matrix** **calculus.**   

In matrix notation [27], the property of *SoftMax trick* has a longer proof, as we first need to calculate the Jacobian of the SoftMax function [28].

If $y_{j}≐\frac{e^{x_{j}}}{\sum_{k\in [K]}e^{x_{k}}}$ , then $\frac{\partial y_{j}}{\partial x_{i}}=\left\{\begin{array}{c} \frac{-e^{x_{j}}\cdot e^{x_{i}}}{{\left(\sum_{k\in [K]}e^{x_{k}}\right)}^{2}}=-y_{i}\cdot y_{j},\;\mathrm{when}\;i\neq j\\ \frac{e^{x_{i}}\cdot \left(\sum_{k\in [K]}e^{x_{k}}\right)-e^{x_{i}}\cdot e^{x_{i}}}{{\left(\sum_{k\in [K]}e^{x_{k}}\right)}^{2}}=y_{i}-y_{i}^{2}=\left(1-y_{i}\right)\cdot y_{i},\\ \;\mathrm{when}\;i=j \end{array}\right.$

The general formula is $\frac{\partial y_{j}}{\partial x_{i}}=\left(\delta_{ij}-y_{i}\right)y_{j}\;.$ Therefore:

$${\left(\frac{\partial y}{\partial x}\right)}_{ij}≐\frac{\partial y_{j}}{\partial x_{i}}=\delta_{ij}y_{j}-y_{i}y_{j}={\left(\mathrm{diag}\left[y\right]\right)}_{ij}-{\left(yy^{⊺}\right)}_{ij}\;.$$

Hence, $\frac{\partial y}{\partial x}=\mathrm{diag}\left[y\right]-yy^{⊺}$ . From the chain rule

$$\frac{\partial \left[SoftCE\left(y^{\circ },x\right)\right]}{\partial x}≐\frac{\partial CE(y^{\circ },y\left(x\right))}{\partial x}={\left(\frac{\partial y}{\partial x}\right)}^{⊺}\cdot \frac{\partial \left[CE\left(y^{\circ },y\right)\right]}{\partial y}\;,\;\mathrm{where}\;\;y\left(x\right)≐SoftMax\left(x\right)$$

and the symmetry of SoftMax Jacobian matrix $\frac{\partial y}{\partial x}$ , we obtain:

(5) $$\begin{array}{ccc} \frac{\partial \left[SoftCE\left(y^{\circ },x\right)\right]}{\partial x} & = & \left(\mathrm{diag}\left[y\right]-yy^{⊺}\right)\left(-y^{\circ }÷y\right)\\ & = & y\underset{1_{K}^{⊺}y^{\circ }=1}{\underset{︸}{{\left(y÷y\right)}^{⊺}y^{\circ }}}-\underset{I_{K}}{\underset{︸}{\mathrm{diag}\left[y÷y\right]}}y^{\circ }=y-y^{\circ } \end{array}$$

   □

While looking at the above two proofs for the Theorem 1, a question can be raised: Is it only the SoftMax function that has *SoftMax trick* property? The answer to this problem can be found in Appendix A. You can understand why the second proof using Jacobian matrix and matrix calculus has been presented here.

## 3. ISBE Functionality

The ISBE functionality is a proposed simplification of the cost function, combining the SoftMax normalization function with the cross-entropy function, hereafter abbreviated as ${\mathrm{CE}}_{all}$ . Its role is to *punish* those calculated probability distributions that significantly differ from the distributions of scores proposed by the *teacher*.

To understand this idea, let us extend the inference diagram for ${\mathrm{CE}}_{all}$ with the backward propagation part for the gradient. We consider this diagram in its separate version, omitting earlier descriptions for the diagram (1):

(6) $$\begin{array}{c} \overset{\mathrm{Loss}\;\mathrm{Inference}\;L}{\overset{︷}{\overset{X}{\to }\begin{array}{c} \mathrm{SoftMax} \end{array}\overset{Y,\;Y^{\circ }}{\to }\begin{array}{c} \mathrm{CrossEntropy} \end{array}\overset{Z}{\to }}}\\ \overset{\mathrm{BackPropagation}}{\overset{︷}{\overset{\bar{X}\overset{Theorem\;1}{\leftarrow }Y-Y^{\circ }}{\leftarrow }\begin{array}{c} \bar{\mathrm{SoftMax}} \end{array}\overset{\bar{Y};\;Y,\;Y^{\circ }}{\leftarrow }\begin{array}{c} \bar{\mathrm{CrossEntropy}} \end{array}\overset{\bar{Z}=1}{\leftarrow }}} \end{array}$$

The meaning of variables $X,Y,Y^{\circ },Z$ and $\bar{Z},\bar{Y},\bar{X}$ appearing in the above diagram (6):

$\begin{array}{cc} X & \mathrm{raw}\;\mathrm{score}\;\mathrm{at}\;\mathrm{the}\;\mathrm{input}\;\mathrm{of}\;\mathrm{the}\;\mathrm{normalization}\;\mathrm{function}\;\mathrm{preceding}\;\text{cross-entropy}\;\mathrm{CE},X\in R^{K},\\ Y & \mathrm{normalization}\;\mathrm{result},\;\text{so-called}\;\mathrm{soft}\;\mathrm{score}\;,Y\in {\left(0,1\right)}^{K},\\ Y^{\circ } & \mathrm{target}\;\mathrm{soft}\;\mathrm{score},\;\mathrm{assigned}\;\mathrm{to}\;\mathrm{the}\;\mathrm{classified}\;\mathrm{example}\;,\\ Z & \mathrm{output}\;\mathrm{of}\;\text{cross-entropy}\;\mathrm{CE}\;,Z\in R,\\ \bar{Z} & \mathrm{formal}\;\mathrm{gradient}\;\mathrm{at}\;\mathrm{the}\;\mathrm{input}\;\mathrm{of}\;\mathrm{the}\;\mathrm{backward}\;\mathrm{propagation}\;\mathrm{algorithm},\;\bar{Z}=1,\\ \bar{Y} & \mathrm{gradient}\;\mathrm{of}\;\text{cross-entropy}\;\mathrm{CE}\;\mathrm{with}\;\mathrm{respect}\;\mathrm{to}\;Y:\;\bar{Y}=\frac{\partial Z}{\partial Y}=-\frac{Y^{\circ }}{Y},\\ \bar{X} & \mathrm{gradient}\;\mathrm{of}\;\text{cross-entropy}\;\mathrm{CE}\;\mathrm{with}\;\mathrm{respect}\;\mathrm{to}\;X:\;\bar{X}\overset{Theorem\;1}{\leftarrow }\left(Y-Y^{\circ }\right)\;. \end{array}$

The key formula here is $\bar{X}\leftarrow \left(Y-Y^{\circ }\right)$ . Its validity comes from the mentioned Theorem 1 which includes the proof for the Formula (4) associated with the *SoftMax trick* property.

The generalized form of this property is given in the Appendix A within the Theorem A1 which includes interesting observations on necessary and sufficient conditions for the *SoftMax trick*.

For instance, the Equation (A2) on the form of the Jacobian of the normalization unit is both a sufficient and necessary condition for its combination with the cross-entropy unit to ensure the equality (A3). Moreover, this condition implies that an activation function with a Jacobian of the *SoftMax* type is a SoftMax function with optional relocation.

Theorem A1 leads us to a seemingly pessimistic conclusion: it is not possible to seek further improvements by changing the activation and at the same time expect the *SoftMax trick* property to hold. Thus, the question arises: what will happen if, along with changing the activation unit, we change the cross-entropy unit to another or even omit it entirely?

In the *ISBE* approach, the aforementioned simplification of the ${\mathrm{CE}}_{all}$ cost function involves precisely omitting the cross-entropy operation in the inference stage and practically omitting all backward operations for this cost function. So what remains? The answer is also an opportunity to decode the acronym *ISBE* again:

1. In the inference phase (I), we normalize the raw score *X* to Y=SoftMax(X) , characterized as a soft score (S).
2. In the backward propagation phase (B), we return an error (E) equal to the difference between the calculated soft score and the target score, i.e., $\bar{X}≐Y-Y^{\circ }$ .

Why can we do this and still consider that in the case of the SoftMax activation function, the value of the gradient transmitted to the network is identical: ${\bar{X}}_{CE_{all}}={\bar{X}}_{ISBE}≐Y-Y^{\circ }$ ?

The answer comes directly from the property ${\bar{X}}_{CE_{all}}=Y-Y^{\circ }$ , formulated in Equation (4), which as it was already mentioned, was proved in the Theorem 1 as the *SoftMax trick* property.

We thus have on the left the following diagram of data and gradient backpropagation through such a unit. On the right, we have its generalization to a ScoreNormalization unit instead of SoftMax unit.

$$\left.\begin{array}{c} \overset{\mathrm{ISBE}\;\mathrm{Inference}}{\overset{︷}{\overset{X}{\to }\begin{array}{c} \mathrm{SoftMax} \end{array}\overset{Y,\;Y^{\circ }}{\to }}}\\ \overset{\bar{ISBE}\;\;\mathrm{BackPropagation}}{\overset{︷}{\overset{\bar{X}\leftarrow Y-Y^{\circ }}{\leftarrow }\begin{array}{c} \mathrm{Subtract} \end{array}\overset{Y,\;Y^{\circ }}{\leftarrow }}} \end{array}\right\}\overset{\mathrm{generalize}}{\to }\left\{\begin{array}{c} \overset{\mathrm{ISBE}\;\mathrm{Inference}}{\overset{︷}{\overset{X}{\to }\begin{array}{c} \mathrm{Score}\;\mathrm{Normalization} \end{array}\overset{Y,\;Y^{\circ }}{\to }}}\\ \overset{\bar{ISBE}\;\;\mathrm{BackPropagation}}{\overset{︷}{\overset{\bar{X}\leftarrow Y-Y^{\circ }}{\leftarrow }\begin{array}{c} \mathrm{Subtract} \end{array}\overset{Y,\;Y^{\circ }}{\leftarrow }}} \end{array}\right.$$

Which activation functions should we reach for in order to test them in the ISBE technique?

1. The SoftMax activation function should be the first candidate for comparison, as it theoretically guarantees behavior comparable to the system containing cross-entropy.
2. Activations should be monotonic so that the largest value of the raw score remains the largest score in the soft score sequence.
3. Soft scores should be within a limited range, e.g., [0,1] as in the case of SoftMax and Sigmoid, or [−1,+1] as for Tanh.
4. The activation function should not map two close scores to distant scores. For example, normalizing a vector of scores by projecting onto a unit sphere in the *p*-th Minkowski norm meets all the above conditions. However, it is not stable around zero. Normalization $\frac{x}{{∥x∥}_{p}}$ maps, for example, two points ϵ,−ϵ distant by $2\cdot {∥\epsilon ∥}_{p}$ to points distant exactly by 2, thus changing their distance $\frac{1}{{∥\epsilon ∥}_{p}}$ times, e.g., a million times, when ${∥\epsilon ∥}_{p}=10^{-6}$ . This operation is known in Pytorch library as normalize function.

The experiments conducted confirm the validity of the above recommendations. The Pytorch library functions SoftMax, sigmoid, tanh, hardsigmoid, hardtanh meet the above three conditions and provide effective classification at a level of effectiveness higher than 99.5% , comparable to CrossEntropy ∘ SoftMax. In contrast, with the function normalize, the optimizer failed to converge on the same MNIST(60K) collection and with the same architectures.

What connects these *good normalization* functions $F:R^{K}\to R^{K}$ , of which two are not even fully differentiable? Certainly, it is the Lipschitz condition occurring in a certain neighborhood of zero [29]:

$$x\in R^{K}{,\;∥x∥}_{p}\leq \epsilon ⟶{∥F\left(x\right)∥}_{p}\leq c{∥x∥}_{p}\;,\;\mathrm{where}\;c\;\mathrm{is}\;a\;\mathrm{certain}\;\mathrm{constant}\;.$$

Note that the Lipschitz condition meets the expectations of the fourth requirement on the above list of recommendations for ISBE. Moreover, we do not expect here that the constant *c* be less than one, i.e., that the function *F* has a narrowing character.

We also need a recommendation for *teachers* preparing class labels, which we represent as vectors blurred around the base vectors of axes $I_{K}=\left[e_{1},\ldots ,e_{K}\right],\;e_{i}\left[j\right]≐\delta_{ij}$ :

1. example blurring value μ , e.g., $\mu =10^{-6}$ :
$${\tilde{e}}_{i}\left[j\right]\leftarrow \left(1-\mu \right)\delta_{ij}+\frac{\mu }{K-1}\left(1-\delta_{ij}\right)$$
2. when the range of activation values is other than the interval [0,1] , we adjust the vector ${\tilde{e}}_{i}$ to the new range, e.g., for tanh the range is the interval (−1,+1) and then the adjustment has the form:
$${\tilde{e}}_{i}\leftarrow 2\cdot {\tilde{e}}_{i}-1,\;i=1,\ldots ,K$$

Finally, let us take a look at the code for the main loop of the program implemented on the Pytorch platform.

1. This is what the code looks like when loss_function is chosen as nn.CrossEntropyLoss:
for (labels,images) in tgen:
     outputs = net(images)
     loss = loss_function(outputs, labels)
     optimizer.zero_grad()
     loss.backward()
     optimizer.step()
2. Now we introduce the ISBE option for SoftMax activation and replace the call for loss function by soft error calculation:
for (labels,images) in tgen:
     outputs = net(images)
     soft_error = SoftMax(outputs) - labels
     optimizer.zero_grad()
     outputs.backward(soft_error)
     optimizer.step()

More options, including the definition of ISBE functionality, can be found in Appendix B. Of course, the above code snippets are only intended to illustrate how easy it is to add the functionality of ISBE to an existing application.

## 4. Experiments

What do we want to learn from the planned experiments? We already know from theory that in the case of the SoftMax activation, we cannot worsen the parameters of the classifier using cross-entropy, both in terms of success rate and learning time.

Therefore, we first want to verify whether theory aligns with practice, but also to check for which normalization functions the ISBE functionality does not degrade the model’s effectiveness compared to ${\mathrm{CE}}_{all}$ .

The learning time $t_{ISBE}$ should be shorter than $t_{CE}$ . Still, to be independent of the specific implementation, we will compare the percentage of the backpropagation time in the total time of inference and backpropagation:

(7) $$\tau ≐\frac{\mathrm{backpropagation}\;\mathrm{time}}{\mathrm{inference}\;\mathrm{time}+\mathrm{backpropagation}\;\mathrm{time}}\times 100\%$$

From many quality metrics, for simplicity, we choose the success rate (also called accuracy), defined as the percentage of correctly classified elements from the test collection MNIST(10K)

(8) $$\alpha =\frac{\mathrm{number}\;\mathrm{of}\;\mathrm{correct}\;\mathrm{classifications}}{\mathrm{size}\;\mathrm{of}\;\mathrm{the}\;\mathrm{test}\;\mathrm{collection}}\times 100\%$$

We want to know how this value changes when we choose different architectures and different activations in the ISBE technique, as well as different options for aggregating cross-entropy over the elements of the training batch.

### 4.1. Experiments with *MNIST* Dataset

Firstly, we evaluate the efficiency of the ISBE idea on the standard MNIST(60K) image collection and the problem of their classification.

We have the following degrees of freedom in our experiments:

1. Two architecture options
  - Architecture $N_{0}$ consists of two convolutions C and two linear units F , of which the last one is a projection from the space of deep feature vectors of dimension 512 to the space of raw scores for each of the K=10 classes:
$$\overset{\mathrm{image}}{\to }I_{28_{yx}}^{1}\;C_{\;3^{k}2^{s}}^{32}\;C_{\;3^{k}2^{s}}^{64}\;D_{20}\;F^{512}\;F^{10}\;\overset{\mathrm{class}\;\mathrm{scores}}{\to }$$
    as by STNN notation [30], for instance
$\left\{\begin{array}{cc} C_{\;3^{k}2^{s}}^{64} & \mathrm{means}\;32\;\mathrm{convolutions}\;\mathrm{with}\;3\;\times \;3\;\mathrm{masks},\;\mathrm{sampled}\;\mathrm{with}\;a\;\mathrm{stride}\;\mathrm{of}\;2,\\ D_{20} & \mathrm{DropOut}—a\;\mathrm{unit}\;\mathrm{zeroing}\;20\%\mathrm{of}\;\mathrm{tensor}\;\mathrm{elements,},\\ F^{512} & a\;\mathrm{linear}\;\mathrm{unit}\;\mathrm{with}\;a\;\mathrm{matrix}\;A\in R^{?\times 512},\\ & \mathrm{here}\;?=64—\mathrm{it}\;\mathrm{is}\;\mathrm{derived}\;\mathrm{from}\;\mathrm{the}\;\mathrm{shape}\;\mathrm{of}\\ & \mathrm{the}\;\mathrm{tensor}\;\mathrm{produced}\;\mathrm{by}\;\mathrm{the}\;\mathrm{previous}\;\mathrm{unit}\;. \end{array}\right.$
  - Architecture $N_{1}$ consists of two blocks, each with three convolutions—it is a purely convolutional network, except for the final projection:
→imageI28yx1C3k32C3k32C5k2s32pD40C3k64C3k64C5k2s64pD40C4k128F10→classscores
    Note that the last convolution in each block has a p requirement for padding, i.e., filling the domain of the image with additional lines and rows so that the image resolution does not change.
2. Three options for reducing the vector of losses in the CrossEntropyLoss element: none, mean, sum.
3. Five options for activation functions used in the ISBE technique:
  - SoftMax: $y_{i}\leftarrow \frac{e^{x_{i}}}{\sum_{j\in [K]}e^{x_{j}}},\;i\in \left[K\right],$
  - Tanh: $y_{i}\leftarrow \frac{e^{x_{i}}-e^{-x_{i}}}{e^{x_{i}}+e^{-x_{i}}},\;i\in \left[K\right],$
  - HardTanh: $y_{i}\leftarrow \left\{\begin{array}{cc} -1 & \mathrm{if}\;x_{i}\leq -1\\ x_{i} & \mathrm{if}\;-1<x_{i}<+1\\ +1 & \mathrm{if}\;+1\leq x_{i} \end{array}\right\},\;i\in \left[K\right],$
  - Sigmoid: $y_{i}\leftarrow \frac{1}{1+e^{-x_{i}}},\;i\in \left[K\right],$
  - HardSigmoid: $y_{i}\leftarrow \left\{\begin{array}{cc} 0 & \mathrm{gdy}\;x_{i}\leq -2\\ \frac{x_{i}+2}{4} & \mathrm{gdy}\;-2<x_{i}<+2\\ +1 & \mathrm{gdy}\;+2\leq x_{i} \end{array}\right\}=\frac{HardTanh(x_{i}/2)+1}{2},\;i\in \left[K\right].$

The results of the experiments, on the one hand, confirm our assumption that the conceptual Occam’s razor, i.e., the omission of the cross-entropy unit, results in time savings τ , and on the other hand, the results are surprisingly positive with an improvement in the metric of success rate α in the case of hard activation functions HardTanh and HardSigmoid . It was observed that only the option of reduction by none behaves exactly according to theory, i.e., the success rate is identical to the model using SoftMax normalization. Options mean and sum for the model with *entropy* are slightly better than the model with *SoftMax*.

The consistency of models in this case means that the number of images incorrectly classified out of 10 thousand is the same. The experiments did not check whether it concerns the same images. A slight improvement, in this case, meant that there were less than a few or a dozen errors, and the efficiency of the model above 99.6% meant at most 40 errors per 10 thousand of test images.

#### 4.1.1. Comparison of Time Complexity

We compare time complexity according to the metric given by the Formula (7).

In the context of time, Table 1 clearly shows that the total timeshare of backpropagation, obviously depending on the complexity of the architecture, affects the time savings of the ISBE technique compared to CrossEntropyLoss—Table 2. The absence of pluses in this table, i.e., the fact that all solutions based on ISBE are relatively faster in the learning phase, is an undeniable fact.

The greatest decrease in the share of backpropagation, over 3% , occurs for the Sigmoid and SoftMax activations. The smallest decrease in architecture $N_{0}$ is noted for the soft (soft) normalization function Tanh and its hard version HardTanh . This decrease refers to cross-entropy without reduction, which is an aggregation of losses calculated for all training examples in a given group into one numerical value.

Inspired by the Theorem A1, which states that the relocation of the SoftMax function preserves the *SoftMax trick* property, we also add data to the Table 1 for the network $N_{1}^{r}$ . This network differs from the $N_{1}$ network only because the normalization unit has a trained relocation parameter. In practice, we accomplish training with relocation for normalization by training with the relocation of the linear unit immediately preceding it. This is done by setting its parameter: bias = True.

As we can see, the general conclusion about the advantage of the ISBE technique in terms of time reduction for the model with the relocation of the normalization function is the same.

#### 4.1.2. Comparison of Classifier Accuracy

Comparison of classifier accuracy and differences in this metric are contained in Table 3 and Table 4. The accuracy is computed according to the Formula (8).

The number of pluses on the side of ISBE clearly exceeds the number of minuses. The justification for this phenomenon requires separate research. Some light will be shed on this aspect by the analysis of learning curves—the variance in the final phase of learning is clearly lower. The learning process is more stable.

In Table 4, we observe that, with the exception of the function SoftMax , which on several images of digits performed worse than the model with cross-entropy, the soft activations have an efficiency slightly or significantly better. However, we are talking about levels of tenths or hundredths of a percent here. The largest difference noted for the option SoftMax was 15-hundredths of a percent, meaning 15 more images correctly classified. Such differences are within the margin of statistical error.

The use of relocation for the normalization function does not provide a clear conclusion—for some models, there is a slight improvement; for others, there is a slight deterioration. It is true that the ISBE functionality with sigmoid activation achieved the best efficiency of 99.69% , but this is only a matter of a few images.

Within the limits of statistical error, we can say that the ISBE functionality gives the same results in recognizing MNIST classes. Its advantages are:

- of decrease time in the total time,
- simplification of architecture, and therefore playing the philosophical role of *Occam’s razor*.

#### 4.1.3. Visual Analysis

Further analysis of the results will be based on the visual comparison of learning curves.

**Figure 1:**
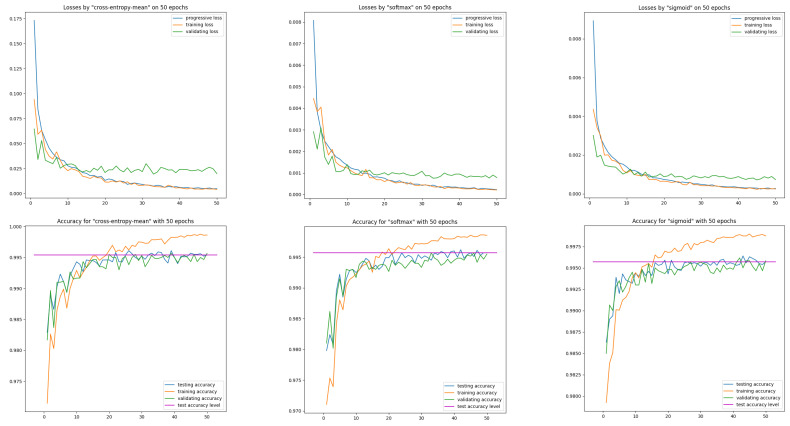
Learning curves on training and validation data for the $N_{1}$ network and three models: cross-entropy-mean, SoftMax, sigmoid. The horizontal reference line represents the accuracy of test data computed after the last epoch.

First, let us see on three models cross-entropy-mean, SoftMax, sigmoid their loss and efficiency curves obtained on training data MNIST(54K) and on data intended solely for model validation MNIST(6K). These two loss curves are calculated after each epoch. We supplement them with a loss curve calculated progressively after each batch of training data (see Figure 1).

Let us note the correct course of the train loss curve with respect to the progressive loss curve—both curves are close. The correct course is also for the validation loss curve—the validation curve from about epoch 30 is below the training curve, maintaining a significant distance. This effect was achieved only after applying a moderate input image augmentation procedure via random affine transformations in the pixel domain.

Correct behavior of learning curves was recorded both for the models with entropy and for models with the ISBE functionality. This also applies to classifier performance curves.

**Figure 2:**
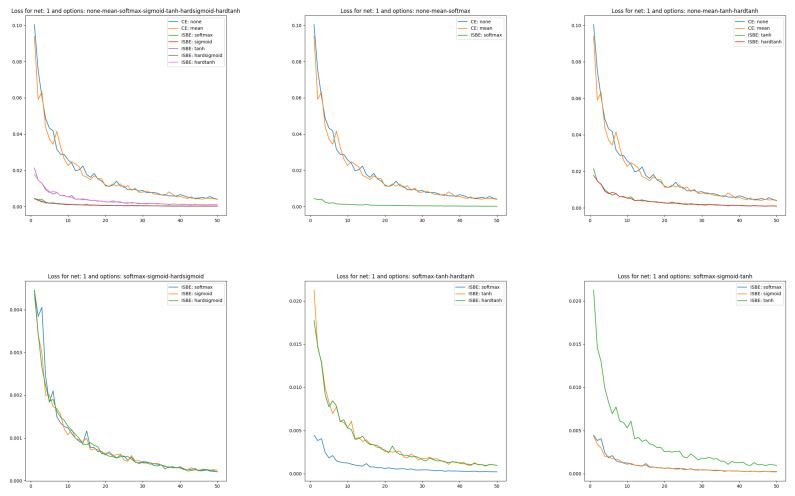
Loss charts of learning in comparisons of CE versus ISBE options. In the first row: (1) all options for loss functions and soft score functions; (2) CE none, CE mean versus SoftMax; (3): CE none, CE mean versus tanh, hardtanh. In the second row: (1) SoftMax versus sigmoid, hardsigmoid; (2) SoftMax versus tanh, hardtanh; (3) SoftMax versus sigmoid, tanh.

1. Curves of loss functions can appear together as long as the type of function is identical, which entails a similar range of variability for loss function values. One might wonder what measure of loss to adopt in the case of ISBE since this technique, in fact, does not calculate loss values. We opt for a natural choice of mean square error for errors in soft scores:
$$L_{ISBE}=MSE\left(Y,Y^{\circ }\right)≐\frac{1}{n_{b}}\cdot {∥Y-Y^{\circ }∥}_{2}^{2}$$
  where $n_{b}$ is the batch size.
  For such defined measures, it turns out that only the option of reduction by summing has a different range of variability, and therefore it is not on the Figure 2.
2. In the case of classifier accuracy, a common percentage scale does not exclude placing all eight curves for each considered architecture. However, due to the low transparency of such a figure, it is also worth juxtaposing different groups of curves of the dependency α(e) . The accuracy α of the classifier MNIST(60K) is calculated on the test set MNIST(10K).

Sets of curves, which we visualize separately for architectures $N_{0}$ , $N_{1}$ are:

- All options for loss functions (3) and soft score functions (5),
- CE none, CE mean, CE sum versus SoftMax,
- CE none, CE mean, CE sum versus tanh, hardtanh,
- SoftMax versus sigmoid, hardsigmoid,
- SoftMax versus tanh, hardtanh,
- SoftMax versus sigmoid, tanh.

**Figure 3:**
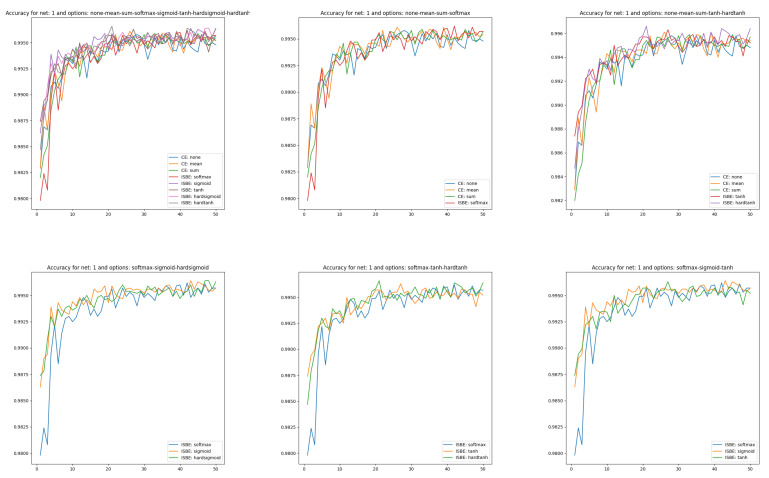
Accuracy charts of learning in comparisons of CE versus ISBE options. In the first row: (1) all options for loss functions and soft score functions; (2) CE none, CE mean versus SoftMax; (3): CE none, CE mean versus tanh, hardtanh. In the second row: (1) SoftMax versus sigmoid, hardsigmoid; (2) SoftMax versus tanh, hardtanh; (3) SoftMax versus sigmoid, tanh.

Due to space constraints, we show learning curves and classifier effectiveness graphs only for architecture $N_{1}$ in Figure 2 and Figure 3.

In Figure 2 we can clearly observe four clusters of models:

- CrossEntropyLoss based with reduction option sum (as out of common range it was not shown),
- CrossEntropyLoss based with reduction options none, and mean,
- ISBE based with normalizations to range [0,1] including functions
SoftMax, Sigmoid, and HardSigmoid ,
- ISBE based with normalizations to range [−1,1] including functions Tanh, and HardTanh .

Within a cluster, the loss curves behave very similarly. Interestingly, the loss curves in ISBE-based clusters tend to the same value greater than zero. In contrast, cross-entropy-based curves also tend to the same limit. However it is clearly greater than ISBE one.

Now, we will pay more attention to test learning curves. We generate test learning curves on the full set of test data MNIST(10K). After each epoch, one point is scored towards the test learning curve. We will show these curves in several comparative contexts.

Accuracy charts of learning (see Figure 3) were obtained to compare cross entropy (CE) performances versus ISBE performance. We have:

- Comparison of CE versus soft options:
  1. all options for loss functions and soft score functions
  2. CE none, CE mean versus SoftMax,
  3. CE none, CE mean versus tanh, hardtanh.
- Comparison of SoftMax versus other soft options:
  1. SoftMax versus sigmoid, hardsigmoid,
  2. SoftMax versus tanh, hardtanh,
  3. SoftMax versus sigmoid, tanh.

In the case of classifier accuracy curves, the variances in the clusters described above are smaller than in the union of clusters. Close to the final epochs, all curves tend to be chaotic within the range of (99.4,99.7) .

**Figure 4:**
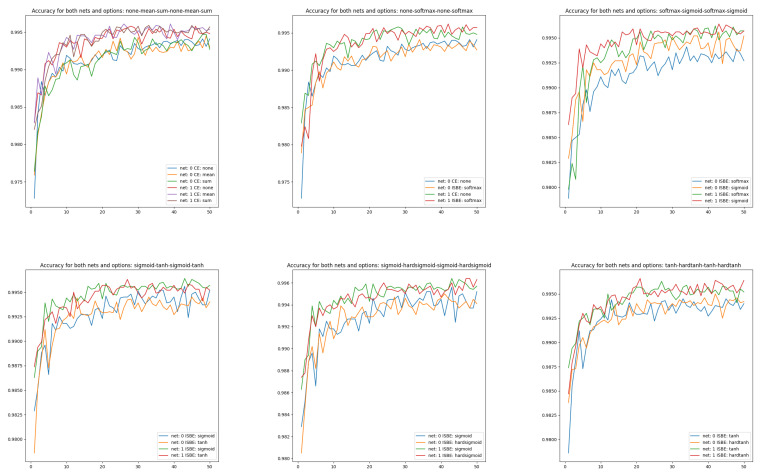
Accuracy charts of learning in comparisons of CE versus ISBE options and architecture $N_{0}$ versus $N_{1}$ . In the first row: (1) CE none, CE mean, CE sum; (2) CE none, SoftMax; (3): SoftMax, sigmoid. In the second row: (1) sigmoid, tanh; (2) sigmoid, hardsigmoid; (3) tanh, hardtanh.

Visualizing the effectiveness of classifiers for different architectures of different complexities, although more obvious, also has research value (see Figure 4):

- CE none, CE mean, CE sum from $N_{0}$ versus CE none, CE mean, CE sum from $N_{1}$ ,
- CE none, SoftMax from $N_{0}$ versus CE none, SoftMax from $N_{1}$ ,
- SoftMax, sigmoid from $N_{0}$ versus SoftMax, sigmoid from $N_{1}$ ,
- sigmoid, tanh from $N_{0}$ versus sigmoid, tanh from $N_{1}$ ,
- sigmoid, hardsigmoid from $N_{0}$ versus sigmoid, hardsigmoid from $N_{1}$ ,
- tanh, hardtanh from $N_{0}$ versus tanh, hardtanh from $N_{1}$ .

Figure 4 shows the better performance of $N_{1}$ compared to $N_{0}$ . Moreover, we can observe slightly more stable behavior for ISBN-based curves than for cross-entropy-based.

### 4.2. Experiments with *CIFAR-10* Dataset

**Figure 5:**
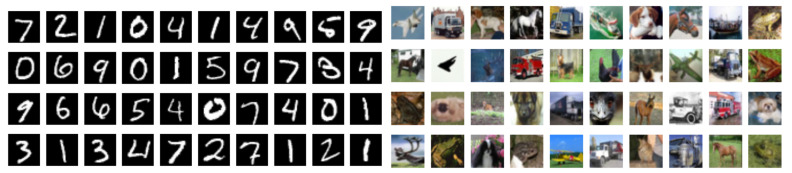
Comparing sample images from MNIST and CIFAR-10 datasets. CIFAR-10 classes: plane, car, bird, cat, deer, dog, frog, horse, ship, truck.

In this subsection, the CIFAR-10—the more demanding than MNIST dataset is considered in the context of ISBE functionality. Moreover, the VGG feature extractor with more than 14 M parameters, i.e., more than 10 times larger model than $N_{1}$ , is joined to make further tests. In Figure 5, we can compare sample images from MNIST dataset and CIFAR-10 dataset. What is immediately observed is the background of objects classified—the uniform black for MNIST and the natural scene in case of CIFAR-10. It is the main reason that despite the almost perfect fit achieved by VGG-16 on the training set CIFAR-10 of 50 thousand images,the best results on the independent testing dataset of 10 thousand images are near 93% . The best results known w CIFAR-10 for all architectures attempted so far are near 95% —about one percent more than the record achieved by human beings.

The architecture VGG-16 was presented by Simonyan and Zisserman in their seminal paper [31], *Very Deep Convolutional Networks for Large-Scale Image Recognition*. *VGG-16* model now serves the community as the universal image feature extractor. Its structure has the following sequential form:

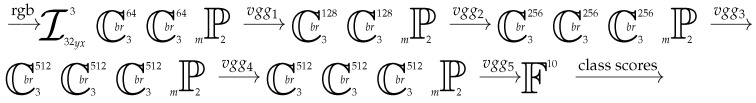



Like for the two architectures $N_{0},N_{1}$ tested for MNIST, the optimizer used for model updates is still AdaM with exponential decay of learning rate with respect to epochs. However, now the initial learning rate is 0.1 , not 0.01 .

**Figure 6:**
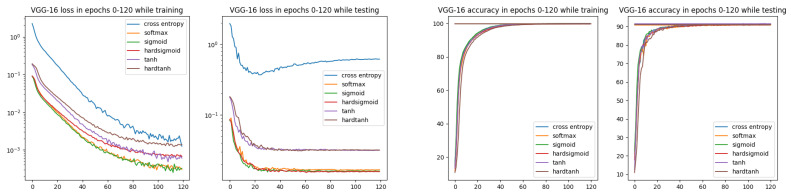
Loss and accuracy charts for VGG-16 architecture and CIFAR-10 dataset. In the loss chart for training, we can observe better convergence for all ISBE options than for cross-entropy.

In Figure 6, we can observe better convergence for all ISBE options than for the cross-entropy. Moreover, during testing, the loss value for CE is slowly increasing, starting at about epoch 30, while for all ISBE options, it is stabilizing on the fixed level.

**Figure 7:**
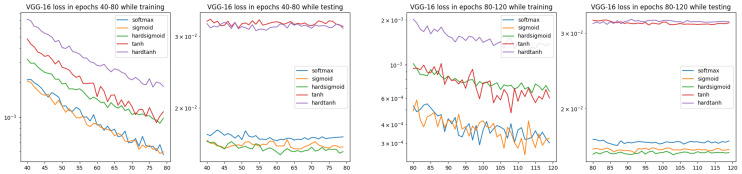
Loss charts for VGG-16 architecture and CIFAR-10 dataset within epochs 40–80 and 80–120 (only ISBE options are shown). During testing, we can observe two clusters for convergence: the sigmoid cluster and the tanh cluster.

From the results presented in Figure 7, it is visible that in the training and testing stages, there are different clusterings for ISBE options:

- In training, there are three groups of ISBE options: {hardtanh}, {tanh, hardsigmoid}, {sigmoid, SoftMax}.
- In testing there are two groups: {tanh, hardtanh} and {SoftMax, sigmoid, hardsigmoid}.

The significant gap between tanh, hardtanh and other ISBE options can be explained by different ranges for the first group and for the second one, i.e., (−1,+1) versus (0,1) . It is not fully clear why in the training stage hardtanh is separate to tanh.

**Figure 8:**
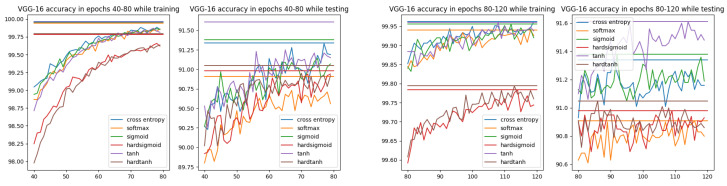
Accuracy charts for VGG-16 architecture and CIFAR-10 dataset within epochs 40–80 and 80–120. Each horizontal line denotes the maximum accuracy for the option of the same color.

In Figure 8, the accuracy for cross-entropy and all ISBE options can be compared. It is observed that hard versions are inferior to others. However, while testing, a slight advantage is achieved by the hyperbolic tangent tanh.

Ultimately, we have bad news on time savings when using autograd interface. Contrary to MNIST experiments where ISBE functionality was implemented by the direct replacement of CE loss in the main learning loop, the CIFAR-10 experiments were using the definition of ISBE_func class being the extension to torch.autograd.Function class. It seems that the general mechanism of interfacing to C++ used by PyTorch in this case, is less efficient than for cross_entropy function. This is perhaps the reason that functionality with fewer operations takes slightly more time while the same functionality without explicit use of autograd mechanism gives always time savings up to 3% .

## 5. Conclusions

Cross-entropy CE as a loss function *owes much to normalization* performed by the SoftMax activation function. In the backward gradient backpropagation phase, only this activation, through perfect linearization, can prevent the explosion or suppression of the gradient originating from CE. What we call the *SoftMax trick*, as a mathematical phenomenon, is explained by the theory presented in the second section and its extension in Appendix A. There is proof that such linearization can only be realized by a function $F:R^{K}\to R^{K}$ with a Jacobian identical to that of the SoftMax function. In turn, such a Jacobian can only be derived for *the dilated and relocated* versions of the SoftMax function.

For further research, there remain practical aspects of a more general Theorem A1 implying that dilated and relocated versions of SoftMax are the only ones having the property of *dilated SoftMax trick*. However, it is quite intuitive that the dilation vector could be used to deal with class unbalanced datasets.

Should we, therefore, celebrate this unique relationship between activation and cost function? In this work, we have shown that it is rather beneficial to use the final effect of the action of this pair, namely the linear value equal to $Y-Y^{\circ }$ , which can be calculated without their participation. This is exactly what the ISBE functionality does—it calculates the soft score vector in the forward step to return in the backward step its error from the target score.

To determine the normalized score, the ISBE functionality can use not only the SoftMax function, as it is not necessary to meet the unity condition, i.e., to ensure a probability distribution as scores of the trained classifier. At least four other activation functions sigmoid, tanh and their hard versions HardSigmoid and HardTanh perform no worse. The choice of these final activations was rather a matter of chance, so researchers face further questions. How do we normalize raw scores and appropriately represent (encode) class labels in relation to this normalization to not degrade the classifier’s results? What properties should such normalization functions have? Experiments suggest that meeting the Lipschitz condition in the vicinity of zero may be one of these properties.

The theoretical considerations presented prove that the ISBE functionality in the process of deep model learning correctly simulates the behavior of the CrossEntropy unit preceded by the SoftMax normalization.

The experiments showed that the ISBE functionality saves the time of forward and backward stages up to 3% , and the effectiveness of the classifier model remains unchanged within the margin of statistical error. Obviously, those gains are strongly dependent on datasets and network architectures.

In turn, a more complex case of integrating ISBE functionality with AD tools (AutoGrad) of a given platform can be solved for PyTorch by copying the proven code from Appendix B. However, as we described in the section on experiments with CIFAR-10, the time savings were consumed by this kind of interfacing to autograd system.

## Figures and Tables

**Table 1:** Comparison of the metric τ , i.e., the percentage share of backpropagation time in the total time with inference. The share $\tau_{CE}$ of cross-entropy with three types of reduction is compared with five functions of soft normalization. The analysis was performed for architectures $N_{0}$ and $N_{1}$ .

Net	Mean	None	Sum	Hsigmoid	Htanh	Sigmoid	SoftMax	Tanh
$N_{0}$	60.61%	59.56%	59.98%	58.31%	58.21%	57.38%	57.45%	59.07%
$N_{1}$	54.89%	53.92%	53.98%	52.68%	52.33%	51.75%	51.95%	52.11%
$N_{1}^{r}$	54.45%	53.92%	54.00%	52.78%	52.30%	51.67%	51.73%	52.11%

**Table 2:** Metric $\Delta \tau ≐\tau_{ISBE}-\tau_{CE}$ , i.e., the decrease in the percentage share of backpropagation time in the total time with inference. The analysis was performed for architectures $N_{0}$ and $N_{1}$ .

Net	CE Loss	Hsigmoid	Htanh	Sigmoid	SoftMax	Tanh
$N_{0}$	mean	−2.30%	−2.40%	−3.23%	−3.16%	−1.54%
$N_{0}$	none	−1.25%	−1.35%	−2.18%	−2.11%	−0.50%
$N_{0}$	sum	−1.67%	−1.77%	−2.60%	−2.53%	−0.92%
$N_{1}$	mean	−2.21%	−2.56%	−3.14%	−2.94%	−2.79%
$N_{1}$	none	−1.24%	−1.59%	−2.17%	−1.97%	−1.82%
$N_{1}$	sum	−1.30%	−1.65%	−2.23%	−2.03%	−1.87%

**Table 3:** In the table, the success rate of three classifiers based on cross-entropy with different aggregation options is compared with the success rate determined for five options of soft score normalization functions. The analysis was performed for architectures $N_{0}$ and $N_{1}$ .

Net	Mean	None	Sum	Hsigmoid	Htanh	Sigmoid	SoftMax	Tanh
$N_{0}$	99.45%	99.41%	99.47%	99.50%	99.50%	99.56%	99.41%	99.45%
$N_{1}$	99.61%	99.58%	99.59%	99.64%	99.66%	99.64%	99.62%	99.63%
$N_{1}^{r}$	99.55%	99.64%	99.64%	99.61%	99.66%	99.69%	99.63%	99.57%

**Table 4:** Change in success rate between models with cross-entropy and models with soft score normalization function. The analysis was performed for architectures $N_{0}$ and $N_{1}$ .

Net	CE Loss	Hsigmoid	Htanh	Sigmoid	SoftMax	Tanh
$N_{0}$	mean	0.05%	0.05%	0.11%	−0.04%	0.00%
$N_{0}$	none	0.09%	0.09%	0.15%	0.00%	0.00%
$N_{0}$	sum	0.13%	0.03%	0.09%	−0.06%	−0.02%
$N_{1}$	mean	0.03%	0.05%	0.03%	0.01%	0.02%
$N_{1}$	none	0.06%	0.08%	0.06%	0.04%	0.05%
$N_{1}$	sum	0.05%	0.07%	0.05%	0.03%	0.04%

## Data Availability

Data and their references are contained within the article.

## Appendix A. Functions Giving the SoftMax Trick for Cross-Entropy

While looking on the two proofs for the Theorem 1 an interesting question arises: is it only the SoftMax function that has *SoftMax trick* property? It seems possible that there are others, as for any differentiable function $F:R^{n}\to R^{n}$ , the starting point for reasoning is the same:

$$\frac{\partial \left[CE(y^{\circ },F\left(x\right))\right]}{\partial x}\overset{y≐F(x)}{=}{\left(\frac{\partial F(x)}{\partial x}\right)}^{⊺}\underset{-y^{\circ }÷y}{\underset{︸}{\frac{\partial \left[CE\left(y^{\circ },y\right)\right]}{\partial y}}}={\left(\frac{\partial F(x)}{\partial x}\right)}^{⊺}\left(-y^{\circ }÷y\right)$$

The following theorem fully characterizes functions that have the *dilated SoftMax trick* property.

**Theorem** **A1** (On the properties of the *SoftMax trick*)**.** *For a differentiable function $F:R^{K}\to R^{K}$ , the following three properties are equivalent:*

1. *F is a generalized SoftMax function if there exist a reference point $c\in R^{K}$ and a dilation vector $d\in R^{K}$ , such that for every $x\in R^{K}$ :*
  (A1)
  y=F(x)=SoftMax(d⊙x−c),
  *where ⊙ operation is the component-wise multiplication.*
2. *F has a dilated SoftMax-type Jacobian, if there exists dilation vector $d\in R^{k}$ , such that for every $x\in R^{K}$ :*
  (A2)
  $$Jacobian\left(F\right)\left(x\right)≐\frac{\partial F(x)}{\partial x}=\mathrm{diag}\left[d⊙y\right]-y{\left(d⊙y\right)}^{⊺}≐\left(D_{y}-yy^{⊺}\right)D_{d}\;,$$
  *where $y=F\left(x\right),\;D_{y}≐\mathrm{diag}\left[y\right],\;D_{d}≐\mathrm{diag}\left[d\right]\;.$
  *
3. *F possesses the dilated SoftMax trick property, if for every target vector $y^{\circ }\in {\left[0,1\right]}^{K},$ and $x\in R^{K}$ its Jacobian matrix $\frac{\partial F(x)}{\partial x}$ satisfies the following equation:*
  (A3)
  $${\left(\frac{\partial F(x)}{\partial x}\right)}^{⊺}\left(-y^{\circ }÷y\right)=D_{d}\left(y-y^{\circ }\right)=d⊙\left(y-y^{\circ }\right)\;,\;where\;y=F\left(x\right)\;.$$

**Proof** **of** **Theorem** **A1.**  We prove the implications in the following order: (2)⟶(3),(3)⟶(2) , (1)⟶(2),(2)⟶(1).

- Proof of implication (2)⟶(3) :
  If the Jacobian $\frac{\partial F(x)}{\partial x}$ of the function *F* is of the *dilated SoftMax* type, then for y=F(x) :
  $$\begin{array}{c} {\left(\frac{\partial F(x)}{\partial x}\right)}^{⊺}\left(-y^{\circ }÷y\right)=D_{d}\left(D_{y}-yy^{⊺}\right)\left(-y^{\circ }÷y\right)\\ =D_{d}\left(y\overset{1_{K}^{⊺}y^{\circ }=1}{\overset{︷}{{\left(y÷y\right)}^{⊺}y^{\circ }}}-\underset{I_{K}}{\underset{︸}{\mathrm{diag}\left[y÷y\right]}}y^{\circ }\right)=D_{d}\left(y-y^{\circ }\right)=d⊙\left(y-y^{\circ }\right) \end{array}$$
- Proof of implication (3)⟶(2) : Denote the axis unit vector *j* by $e_{j}\in R^{K}$ . Then ${\left(e_{j}\right)}_{i}=\delta_{ij}$ . Substitute into property (A3) the target score vector $y^{\circ }≐e_{j}$ . Then
  $$d_{i}\left(y_{i}-{\left(e_{j}\right)}_{i}\right)={\left(\frac{\partial F(x)}{\partial x_{i}}\right)}^{⊺}\left(-e_{j}÷y\right)=\sum_{k\in [K]}\frac{\partial y_{k}}{\partial x_{i}}\cdot \left(\frac{-\delta_{kj}}{y_{k}}\right)=\frac{\partial y_{j}}{\partial x_{i}}\cdot \left(\frac{-1}{y_{j}}\right)$$
  Therefore $\frac{\partial y_{j}}{\partial x_{i}}=d_{i}\cdot \left({\left(e_{j}\right)}_{i}-y_{i}\right)y_{j}\overset{{\left(e_{j}\right)}_{i}=\delta_{ij}}{=}d_{i}\cdot \left(\delta_{ij}-y_{i}\right)y_{j}$ . Swapping *i* with *j* we obtain: $\frac{\partial y_{i}}{\partial x_{j}}=\left(\delta_{ji}-y_{i}\right)\cdot d_{j}$ .
  Thus,
  $$\frac{\partial y}{\partial x}=\left(\mathrm{diag}\left[y\right]-yy^{⊺}\right)⊙d=\left(D_{y}-yy^{⊺}\right)D_{d}=\mathrm{diag}\left[d⊙y\right]-y{\left(d⊙y\right)}^{⊺}\;.$$
- Proof of implication (1)⟶(2) :
  If $y_{j}≐\frac{e^{d_{j}x_{j}-c_{j}}}{\sum_{k\in [K]}e^{d_{k}x_{k}-c_{k}}}$ , then
  $\frac{\partial y_{j}}{\partial x_{i}}=\left\{\begin{array}{c} \frac{-e^{d_{j}x_{j}-c_{j}}\cdot e^{d_{i}x_{i}-c_{i}}\cdot d_{i}}{{\left(\sum_{k\in [K]}e^{d_{k}x_{k}-c_{k}}\right)}^{2}}=-\left(d_{i}y_{i}\right)\cdot y_{j},\;\mathrm{when}\;i\neq j\\ \frac{d_{i}\cdot e^{d_{i}x_{i}-c_{i}}\cdot \left(\sum_{k\in [K]}e^{d_{k}x_{k}-c_{k}}\right)-e^{d_{i}x_{i}-c_{i}}\cdot e^{d_{i}x_{i}-c_{i}}\cdot d_{i}}{{\left(\sum_{k\in [K]}e^{d_{k}x_{k}-c_{k}}\right)}^{2}}\\ =d_{i}\left(y_{i}-y_{i}^{2}\right)=\left(1-y_{i}\right)\cdot \left(d_{i}y_{i}\right),\;\mathrm{when}\;i=j \end{array}\right.$
  The general formula is $\frac{\partial y_{j}}{\partial x_{i}}=\left(\delta_{ij}-y_{j}\right)\left(d_{i}y_{i}\right)\;.$ Therefore:
  $${\left(\frac{\partial y}{\partial x}\right)}_{ji}≐\frac{\partial y_{j}}{\partial x_{i}}=\delta_{ij}\left(d_{i}y_{i}\right)-y_{j}\left(d_{i}y_{i}\right)={\left(\mathrm{diag}\left[d⊙y\right]\right)}_{ji}-{\left(y{\left(d⊙y\right)}^{⊺}\right)}_{ji}\;.$$
  Hence, $\frac{\partial y}{\partial x}=\mathrm{diag}\left[d⊙y\right]-y{\left(d⊙y\right)}^{⊺}=\left(D_{y}-yy^{⊺}\right)D_{d}$
- Proof of implication (2)⟶(1) :
  If $\frac{\partial y_{i}}{\partial x_{i}}=\left(\left(1-y_{i}\right)\cdot y_{i}\right)\cdot d_{i}$ and $\frac{\partial y_{j}}{\partial x_{i}}=-y_{j}y_{i}d_{i}$ then the diagonal of the Jacobian matrix gives us differential Equations [32], from which we can determine the general form of the function $y_{i}\left(x\right)$ , i∈[K]:
  $$\begin{array}{c} \frac{\partial y_{i}}{\left(1-y_{i}\right)\cdot y_{i}}=d_{i}\partial x_{i}⟶\int \left(\frac{1}{y_{i}}+\frac{1}{1-y_{i}}\right)\partial y_{i}=\int d_{i}\partial x_{i}\\ ⟶\log_{e}\frac{y_{i}}{1-y_{i}}=d_{i}x_{i}+C\left(k\neq i\right)⟶y_{i}=\frac{e^{d_{i}x_{i}}}{\underset{z_{i}}{\underset{︸}{e^{d_{i}x_{i}}+e^{-C(k\neq i)}}}}\\ ⟶y_{i}=\frac{e^{d_{i}x_{i}}}{z_{i}},\;\mathrm{where}\;z_{i}=z_{i}\left(x_{1},\cdots ,x_{K}\right)>0\\ ⟶\log_{e}z_{i}=d_{i}x_{i}-\log_{e}y_{i} \end{array}$$
  Now we calculate the partial derivatives $\frac{\partial \log_{e}z_{j}}{\partial x_{i}}$ . If i≠j , then
  $$\frac{\partial \left[\log_{e}z_{j}\right]}{\partial x_{i}}=\frac{\partial \left[d_{j}x_{j}-\log_{e}y_{j}\right]}{\partial x_{i}}=-\frac{1}{y_{j}}\overset{-y_{j}y_{i}d_{i}}{\overset{︷}{\frac{\partial y_{j}}{\partial x_{i}}}}=y_{i}d_{i}$$
  For i=j , the result is the same:
  $$\frac{\partial \left[\log_{e}z_{i}\right]}{\partial x_{i}}=\frac{\partial \left[d_{i}x_{i}-\log_{e}y_{i}\right]}{\partial x_{i}}=d_{i}-\frac{1}{y_{i}}\overset{\left(1-y_{i}\right)y_{i}d_{i}}{\overset{︷}{\frac{\partial y_{i}}{\partial x_{i}}}}=d_{i}-\frac{\left(1-y_{i}\right)y_{i}d_{i}}{y_{i}}=y_{i}d_{i}$$
  Therefore, for any j∈[K] , we have *K* equalities: $\frac{\partial \left[\log_{e}z_{j}\right]}{\partial x_{i}}=d_{i}y_{i}=\frac{\partial \left[\log_{e}z_{1}\right]}{\partial x_{i}}$ , i∈[K] . This means that vector fields for each pair of functions $\log_{e}z_{j}$ and $\log_{e}z_{1}$ are identical.
  Integrating these fields yields the same function up to a constant $c_{j}$ : $\log_{e}z_{j}=\log_{e}z_{1}+c_{j},$ j∈[K] . Consequently, $z_{j}=z_{1}\cdot e^{c_{j}}$ , j∈[K] , and therefore $y_{j}=\frac{e^{d_{j}x_{j}}}{z_{1}e^{c_{j}}}=\frac{e^{d_{j}x_{j}-c_{j}}}{z_{1}}$ . From the *unity* condition, we can now determine the value of $z_{1}$ :
  $$1=\sum_{k\in [K]}y_{k}=\sum_{k\in [K]}\frac{e^{d_{k}x_{k}-c_{k}}}{z_{1}}⟶z_{1}=\sum_{k\in [K]}e^{d_{k}x_{k}-c_{k}}⟶y_{j}=\frac{e^{d_{j}x_{j}-c_{j}}}{\sum_{k\in [K]}e^{d_{k}x_{k}-c_{k}}}\;.$$

   □

Note that the above theorem excludes the functions Sigmoid, Tanh and HardSigmoid, HardTanh from the group of functions for which we can apply the SoftMax trick. Namely, in the vector version, all these functions, none of them can be considered as the special form of the generalized SoftMax function. It is obvious fact, but to give a formal reason, we observe that all those functions operate on each component of vector *x* independently, i.e., the result $y_{i}$ depends only on argument $x_{i}$ . In the generalized SoftMax function, $y_{i}$ depends on all arguments $x_{1},\cdots ,x_{n}$ .

## Appendix B. ISBE Functionality in PyTorch

### Appendix B.1. Testing Soft Options-Direct Way

ISBE functionality can be introduced to our training procedures in many ways.

1. The simplest way of replacing the call of the cross-entropy function is by calling SoftMax and, after, subtracting target hot vectors or their soften versions, calling the backward for the net output:
  for (labels,images) in tgen:
       outputs = net(images)
       soft_error = SoftMax(outputs) - labels
       optimizer.zero_grad()
       outputs.backward(soft_error)
       optimizer.step()
2. If we want to test more options and compare them with cross-entropy, the loop code will extend a bit:
  for (labels,images) in tgen:
       outputs = net(images)
       if no_cross_entropy:
           if soft_option=="SoftMax":
               soft_error = SoftMax(outputs) - labels
           if soft_option=="tanh":
               soft_error = tanh(outputs) - (2.*labels-1.)
           elif soft_option=="hardtanh":
               soft_error = hardtanh(outputs) - (2.*labels-1.)
           elif # …
                # next options
           optimizer.zero_grad()
           outputs.backward(soft_error)
       else:
           loss = loss_function(outputs, labels)
           optimizer.zero_grad()
           loss.backward()
       optimizer.step()
3. If we prefer to have a visually shorter loop, then by introducing the variable soft_function and extending the class DataProvider with matching target labels for a given soft option, we finally obtain a compact form:
  for (labels,images) in tgen:
       outputs = net(images)
       if no_cross_entropy:
           soft_error = soft_function(outputs) - labels
           optimizer.zero_grad()
           outputs.backward(soft_error)
       else:
           loss = loss_function(outputs, labels)
           optimizer.zero_grad()
           loss.backward()
       optimizer.step()
4. However, if we want to register ISBE functionality as torch.autograd.Function then we have to follow the instruction of PyTorch on this kind registration. The effect is described in the next subsection.

### Appendix B.2. *ISBE* Functionality with Automatic Differentiation

In order to make ISBE_func callable in both inference and backpropagation stage we have to define three static methods in extension of class torch.autograd.Function:

class ISBE_func(torch.autograd.Function):

  @staticmethod

  def forward(...)

   <body of forward>

  @staticmethod

  def setup_context(...)

   <body of setup_context>

  @staticmethod

  def backward(...)

   <body of backward>

The whole job is performed in the body of forward static method. Other two methods simply switch tensors:

1. Body of forward:
  def forward(raw_scores, labels,
              options=dict(soft=’SoftMax’, num_classes=10, eps=1e-8)):
       K = options[’num_classes’]
       eps = options[’eps’]; soft_option = options[’soft’]
       one_hots =  torch.nn.functional.one_hot(\
                   labels, num_classes=K)*(1-K*eps)+eps)
       if soft_option==’SoftMax’:
           soft_scores = torch.nn.functional.SoftMax(raw_scores,dim=1)
           target_scores = one_hots
       elif soft_option==’sigmoid’:
           soft_scores = torch.nn.functional.sigmoid(raw_scores)
           target_scores = one_hots
       elif soft_option==’hardsigmoid’:
           soft_scores = torch.nn.functional.hardsigmoid(raw_scores)
           target_scores = one_hots
       elif soft_option==’tanh’:
           soft_scores = torch.nn.functional.tanh(raw_scores)
           target_scores = 2.*one_hots-1.
       elif soft_option==’hardtanh’:
           soft_scores = torch.nn.functional.hardtanh(raw_scores)
           target_scores = 2.*one_hots-1.
       soft_scores.requires_grad = False
       soft_errors = soft_scores - target_scores
       mse_soft = torch.mean(soft_errors**2)
       return mse_soft, soft_errors, soft_scores
2. Body of setup_context:
  def setup_context(ctx, inputs, output):
      raw_scores, labels, options = inputs
      mse_soft, soft_errors, soft_scores = output
      ctx.set_materialize_grads(False)
      ctx.soft_errors = soft_errors
3. Body of backward:
  def backward(ctx, grad_mse, grad_errors, grad_scores):
      return ctx.soft_errors, None, None

We cannot directly call the forward static function. We have to use apply method.

def isbe_func_(raw_scores, labels,

               options=dict(soft=’SoftMax’, num_classes=10, eps=1e-8)):

  return ISBE_func.apply(raw_scores, labels, options)

We could also simplify the use of options if there is a global object ‘ex’ which includes its reference:

isbe_func = lambda raw_scores,labels:\

  isbe_func_(raw_scores, labels, options=ex.loss_options)[0]

Instead of a method backward on PyTorch tensor we could use its wrapper isbe_backward:

isbe_backward = lambda soft_error: soft_error.backward()

Finally we can also hide the options for the F.cross_entropy function:

tones_ = torch.ones(ex.batch_size).to(ex.device)

cross_entropy_func = lambda x,t:\

  F.cross_entropy(x,t,reduction=ex.loss_options[’reduction’],

                  label_smoothing=ex.loss_options[’label_smoothing’])

ce_backward = lambda loss: loss.backward(tones_[:loss.size(0)])\

   if ex.loss_options[’reduction’]==’none’ else loss.backward()
